# Predictive Factors for Efficacy and Safety of Prophylactic Theophylline for Extubation in Infants with Apnea of Prematurity

**DOI:** 10.1371/journal.pone.0157198

**Published:** 2016-07-07

**Authors:** Tomoko Kondo, Yuki Kondo, Yuji Orita, Fumi Mitarai, Yoichi Ishitsuka, Mitsuru Irikura, Yoshihiro Shimodozono, Tsutomu Douchi, Yasuo Takeda, Tetsumi Irie

**Affiliations:** 1 Department of Clinical Chemistry and Informatics, Graduate School of Pharmaceutical Sciences, Kumamoto University, Kumamoto, Japan; 2 Department of Clinical Pharmacy and Pharmacology, Kagoshima University Hospital, Kagoshima, Japan; 3 Department of Obstetrics and Gynecology, Faculty of Medicine, Kagoshima University, Kagoshima, Japan; 4 Laboratory of Evidence-based Pharmacotherapy, College of Pharmaceutical Sciences, Daiichi University, Fukuoka, Japan; 5 Laboratory of Drug Informatics, Department of Pharmaceutical Sciences, Kyusyu University of Health and Welfare, Nobeoka, Japan; 6 Center for Clinical Pharmaceutical Sciences, Faculty of Pharmaceutical Sciences, Kumamoto University, Kumamoto, Japan; Mario Negri Institute for Pharmacology Research, ITALY

## Abstract

**Purpose:**

This study aimed to evaluate predictive factors involved in efficacy and safety in Japanese infants who received theophylline therapy to prevent apnea of prematurity (AOP) after weaning from mechanical ventilation.

**Methods:**

We retrospectively reviewed the medical records of infants who were administered intravenous aminophylline (theophylline ethylenediamine) for AOP at the neonatal intensive care unit, Kagoshima University Hospital, Japan, between January 2009 and June 2013.

**Results:**

A total of 100 infants were evaluated as two separate groups in terms of efficacy and safety of theophylline. Sixty-seven (67.0%) infants had effective theophylline therapy. Multivariate logistic regression analysis showed that gestational age at birth was significant, with an odds ratio of 0.59 (p < 0.001). Receiver operating characteristic analysis showed that the cut-off value was 31.1 weeks old for predicting the efficacy of theophylline (specificity, 66.7%; sensitivity, 86.6%; p < 0.001; area under the curve, 0.750; 95% confidence interval, 0.45–0.74). Adverse reactions were identified in 21 (21.0%) infants. Multivariate logistic regression analysis showed that the number of days of theophylline administration from birth was associated with an increased risk of adverse reactions after theophylline administration (p = 0.01).

**Conclusions:**

Physicians need to be aware of the possibility that theophylline fails to produce therapeutic effects for extubation in infants aged less than 31.1 weeks old, and adverse reactions can easily develop when theophylline is administered soon after birth.

## Introduction

Apnea of prematurity (AOP) is defined as the cessation of breathing for greater than 20 seconds or cessation of breathing for less than 20 seconds accompanied by bradycardia or cyanosis [1, 2]. The incidence of AOP is 25% in neonates who weigh more than 2500 g at birth, and it approaches 84% for those less than 1000 g at birth [3]. AOP can spontaneously occur and appears to be due to immaturity of the infant’s neurological and respiratory systems [3]. While appropriate respiratory support is essential in AOP, treatment of AOP is fairly limited and pharmacotherapy is important. First-line treatment for AOP consists of methylxanthines [4–6].

Theophylline, one of the methylxanthine drugs, is used for infants with AOP. However, theophylline has a narrow therapeutic range [7], and it sometimes leads to adverse events and toxicity, such as vomiting, tachycardia, jitteriness, and irritability [8, 9].

Generally, proper use of medicine is important, and appropriate adjustment of the dose to maintain the therapeutic range is essential for safe and effective use of methylxanthines. However, Skouroliakou et al. reported that serum theophylline concentrations that fell within the recommended therapeutic range in the majority of cases were not significantly associated with apnea events in preterm infants [10]. This finding suggests that the efficacy of theophylline might be due to other causes than its concentration.

Theophylline has also been used to prevent AOP after weaning from mechanical ventilation [11]. Some investigators have reported that, despite administration of theophylline, weaning from mechanical ventilation may be prolonged [12]. Even if extubation is achieved, frequent episodes of apnea may occur in association with respiratory failure, leading to reintubation. However, few reports have been published concerning the predictive factors associated with not only efficacy, but also safety of prophylactic theophylline for extubation in preterm infants [13].

In this study, we aimed to investigate the clinical features of infants who received theophylline therapy to prevent AOP after weaning from mechanical ventilation and evaluated predictive factors involved in its efficacy and safety.

## Materials and Methods

### Infants’ characteristics

**Fig 1:**
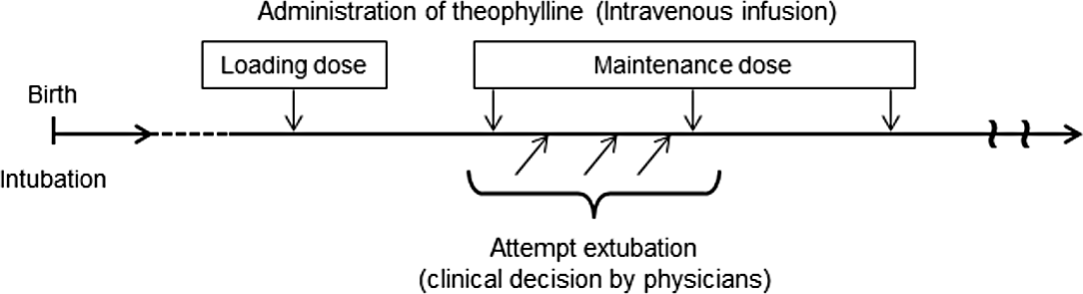
Basic protocol of theophylline administration when weaning from mechanical ventilation.

We performed a retrospective analysis of medical records of Japanese infants who were administered intravenous aminophylline (theophylline ethylenediamine) for AOP. Data were collected at the neonatal intensive care unit, Kagoshima University Hospital, Japan, between January 2009 and June 2013. The methods of theophylline administration are shown in Fig 1.

Eligible infants were those who had been administered theophylline during mechanical ventilation. Of the original 139 infants, 36 were excluded from the analysis because they did not choose to use mechanical ventilation, and two were excluded because they were lacking data for theophylline administration because of changing hospitals. One infant who had difficulty with extubation was excluded because he had to use mechanical ventilation owing to Wilson–Mikity syndrome.

### Data collection

The following data were collected: sex; gestational age at birth; Apgar scores at 1 and 5 minutes; presence/absence of oxygen or enteral nutrition support; presence of a single or twin fetus; body weight when theophylline administration was started; total number of apneas for 5 days after extubation; laboratory test results at birth; postconceptional age when theophylline administration was started; duration and time of theophylline administration; loading and maintenance dose of theophylline; need for additional treatment for AOP after theophylline administration, such as use of continuous positive airway pressure, reintubation, or respiratory stimulants; and adverse reactions.

With regard to the pharmacokinetic parameters of theophylline, total body clearance (CL), volume of distribution (Vd), and the elimination rate constant (k_el_) were estimatedfrom population pharmacokinetic data analysis of theophylline by Fukuda et al [14]. These pharmacokinetic parameters were as follows: CL (mL/h) = [6.98 × body weight (kg)^2.17^ + 0.244 × PCA (weeks)] × 1.24^oxygen support (0 for without oxygen support, 1 for with oxygen support)^, Vd (L) = 0.492 × body weight (kg), and k_el_ (1/h) = CL/Vd. Based on CL, Vd, and kel, the initial theophylline concentration immediately after the bolus injection (C_0_), maximum concentration (Cmax), minimum concentration (Cmin), mean concentration (Cmean), and the area under the curve (AUC) were calculated using y Q-flex software provided by The Japanese Society of Therapeutic Drug Monitoring (available from http://www.asahi-net.or.jp/~ui6m-sby/). Additionally, we conducted a validation exercise to evaluate our prediction. Five neonates were studied among patients who were assigned to an ongoing prospective study to examine the PK parameters of prophylactic theophylline (aminophylline) for extubation in preterm infants at Kagoshima University Hospital for validation analysis. We validated the prediction of theophylline Cmin using the mean prediction error and mean absolute prediction error. The results of the validation analysis are shown in S1 Table. Generally, if the mean and 95% confidence interval (CI) of the mean absolute prediction error are sufficiently small compared with the therapeutic range, the predictive performance of the model is superior. As a result, we decided that our prediction was relatively precise.

### Outcomes

For effectiveness, the infants were classified into two groups: the “effective” group and the “ineffective” group. “Effective” was defined in this study as less than five episodes of apnea per day for 5 days after extubation without reintubation and doxapram administration, which were used in patients who were unresponsive to methylxanthines.

The safety of theophylline was assessed when physicians noted adverse reactions after the start of theophylline treatment. The infants were classified into two groups: the adverse reaction (+) group and the adverse reaction (−) group.

### Statistical analysis

All analyses were conducted using anonymized data. Continuous variables are expressed as mean ± standard deviation. Data of the two groups were compared using the unpaired t test, Fisher’s exact test, or the Mann–Whitney U test. Multivariate logistic regression analysis was used to test the outcomes from univariate logistic regression analysis. Odds ratios and 95% CIs are presented. Parameters that showed a correlation (p < 0.2) in univariate analysis were included in multivariate analysis. A receiver operating characteristic (ROC) curve for the factor of efficacy of theophylline was created with the AUC to determine the cut-off values. A two-tailed p value < 0.05 was considered to indicate statistical significance. Statistical analysis was performed using JMP 11 (SAS Institute Inc., Cary, NC, USA).

This study was approved by the Ethics Committee of Kagoshima University (no. 272) and Kumamoto University (no. 649). All analyses were conducted using anonymized data.

## Results

### Effectiveness of theophylline

A total of 100 infants were analyzed. Sixty-seven infants were classified as the effective group, with an incidence of 67.0%. Thirty-three (33.0%) infants were classified as the ineffective group. In the ineffective group, 28 of these infants had more than six episodes of apnea per day after extubation, 10 were switched to doxapram, and seven required reintubation.

Comparison of the background variables between the effective and ineffective groups is shown in Table 1. There were no significant differences in predicted concentrations of theophylline, such as Co, Cmax, Cmin, and Cmean, between the two groups. The number of twin fetuses, gestational age at birth, body weight and postconceptional age when theophylline administration was started, serum alanine aminotransferase levels, total protein levels, and calcium levels were significantly different between the two groups.

**Table 1:** Characteristics of infants who were classified by the efficacy of theophylline.

	Effective	Ineffective	p value
Case (male/female)	67 (35/32)	33 (16/17)	0.83
Twin fetus	6	9	<0.05
Gestational age (weeks)	33.5 (2.4)	31.00 (2.1)	<0.005
Apgar score 1^a^	6.9 (1–9)	6.7 (1–10)	0.58
Apgar score 5^a^	8.3 (4–10)	8.3 (6–10)	0.98
Body weight when theophylline administration was started (g)	1813.2 (503.1)	1415.5 (491.5)	<0.001
Postconceptional age when theophylline administration was started (weeks)	33.9 (2.4)	31.4 (2.0)	<0.001
Loading dose of theophylline (mg/kg)	4.82 (1.00)	4.93 (0.54)	0.73
Maintenance dose of theophylline (mg/kg/d)	1.41 (0.59)	1.31 (0.39)	0.15
CL (L/kg/h)	0.021 (0.004)	0.018 (0.004)	<0.005
Predicted concentration of theophylline			
Co (mg/L)	9.85 (1.78)	10.02 (1.10)	0.60
Cmax (mg/L)	11.38 (2.81)	11.65 (2.70)	0.65
Cmin (mg/L)	6.32 (2.48)	6.74 (2.21)	0.44
Cmean (mg/L)	8.04 (2.23)	8.83 (2.37)	0.10
Number of days of theophylline administration from birth	2.76 (4.09)	3.06 (2.03)	0.69
Duration of theophylline administration (days)	3.43 (5.39)	4.03 (4.21)	0.58
Frequency of theophylline administration	10.9	12.3	0.63
WBC (/μL)	12280.46 (7274.34)	10731.67 (5024.57)	0.25
RBC (10^4^/μL)	454.09 (79.29)	472.70 (93.46)	0.29
Hemoglobin (g/dL)	17.13 (3.03)	17.42 (2.83)	0.63
Hematocrit (%)	49.77 (8.60)	50.38 (8.29)	0.73
Platelets (10^4^/μL)	21.51 (6.36)	22.56 (5.81)	0.42
AST (IU/L)	53.53 (49.29)	53.97 (23.93)	0.96
ALT (IU/L)	7.79 (13.62)	4.79 (1.93)	<0.001
LDH (IU/L)	563.00 (249.58)	599.18 (203.20)	0.47
ALP (IU/L)	706.81 (266.99)	788.47 (222.97)	0.15
ChE (IU/L)	223.50 (44.23)	224.60 (50.82)	0.92
Total protein (g/dL)	4.66 (0.74)	4.22 (0.65)	<0.05
Total bilirubin (mg/dL)	2.60 (0.92)	2.48 (0.67)	0.51
Direct bilirubin (mg/dL)	0.97 (0.27)	0.97 (0.25)	0.97
GT (IU/L)	272.53 (189.93)	237.56 (130.41)	0.34
BUN (mg/dL)	9.12 (4.97)	8.90 (4.48)	0.83
Serum creatinine (mg/dL)	0.62 (0.18)	0.63 (0.14)	0.76
Amylase (IU/L)	8.38 (4.67)	9.11 (3.61)	0.55
CK (IU/L)	342.69 (163.55)	329.39 (175.25)	0.74
Ca (mg/dL)	8.91 (0.77)	8.54 (0.84)	<0.05
Na (mEq/L)	137.77 (3.00)	137.68 (2.94)	0.89
K (mEq/L)	5.19 (0.87)	5.25 (1.06)	0.79
Cl (mEq/L)	104.80 (3.56)	105.94 (3.10)	0.12
Serum albumin (g/dL)	3.03 (0.40)	2.95 (0.35)	0.49

^a^Data are expressed as median (range).

**Fig 2:**
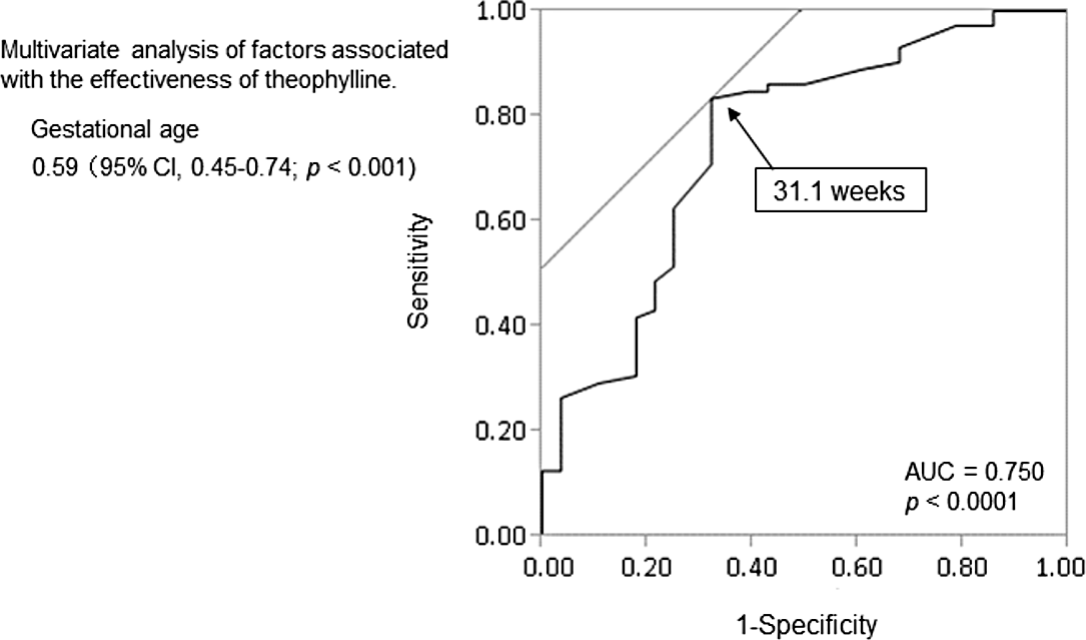
Receiver operating characteristic curve for predicting efficacy of theophylline with gestational age.

In multivariate logistic regression analysis, gestational age at birth was significant, with an odds ratio of 0.59 (95% CI, 0.45–0.74; p < 0.001). The ROC curve of gestational age as a significant predictor of efficacy showed an AUC of 0.750. The cut-off value of 31.1 weeks old (Fig 2) had a specificity of 66.7% and a sensitivity of 86.6% in predicting efficacy of theophylline.

### Safety

Adverse reactions were identified in 21 (21.0%) infants. Among them, 15 had abdominal fullness, four had tachypnea, two had vomiting, one had hypoglycemia, and one had nervous irritability.

Comparison of the background variables between the adverse reaction (+) group and the adverse reaction (−) group is shown in Table 2. The number of days of theophylline administration from birth was significantly lower and serum amylase levels were significantly higher in the adverse reaction (+) group than in the adverse reaction (−) group.

**Table 2:** Characteristics of infants who were classified by the appearance of adverse reactions.

	Adverse reaction (+)	Adverse reaction (-)	p value
Case (male/female)	21 (11/10)	79 (40/39)	1
Twin fetus	1	14	0.18
Gestational age (weeks)	33.0 (2.7)	32.6 (2.5)	0.49
Apgar score 1^a^	7.0 (3–10)	6.8 (1–9)	0.64
Apgar score 5^a^	8.3 (4–10)	8.3 (6–10)	0.95
Body weight when theophylline administration was started (g)	1788.7 (494.3)	1653.5 (533.7)	0.30
Postconceptional age when theophylline administration was started (weeks)	33.3 (2.6)	33.0 (2.5)	0.62
Loading dose of theophylline (mg/kg)	5.08 (0.41)	4.92 (0.55)	0.22
Maintenance dose of theophylline (mg/kg/d)	1.42 (0.25)	1.44 (0.51)	0.91
CL (L/kg/h)	0.020 (0.004)	0.019 (0.004)	0.27
Predicted concentration of theophylline			
Co (mg/L)	10.32 (0.83)	9.80 (1.70)	0.13
Cmax (mg/L)	11.88 (2.79)	11.39 (2.71)	0.46
Cmin (mg/L)	6.73 (2.22)	6.72 (1.97)	0.98
Cmean (mg/L)	8.29 (2.13)	8.30 (2.33)	0.99
AUC (mg・hr/L)	1458.5	952.5	0.21
Number of days of theophylline administration from birth	1.81 (1.14)	3.14 (3.86)	<0.05
Duration of theophylline administration (days)	4.57 (7.15)	3.38 (4.21)	0.36
Frequency of theophylline administration	14.8	8.6	0.25
WBC (/μL)	11848.95 (6239.74)	11748.20 (6689.86)	0.95
RBC (10^4^/μL)	482.10 (59.29)	454.42 (88.27)	0.18
Hemoglobin (g/dL)	18.17 (2.22)	16.97 (3.06)	0.09
Hematocrit (%)	52.02 (6.44)	49.43 (8.79)	0.20
Platelets (10^4^/μL)	21.05 (5.54)	22.07 (6.27)	0.50
AST (IU/L)	59.29 (24.54)	52.17 (45.68)	0.52
ALT (IU/L)	6.90 (5.12)	6.76 (12.31)	0.96
LDH(IU/L)	581.86 (119.90)	573.23 (255.60)	0.88
ALP (IU/L)	711.29 (204.19)	739.53 (255.60)	0.65
ChE (IU/L)	222.16 (35.23)	224.35 (48.57)	0.85
Total protein (g/dL)	4.68 (0.66)	4.46 (0.74)	0.23
Total bilirubin (mg/dL)	2.48 (0.36)	2.58 (0.92)	0.62
Direct bilirubin (mg/dL)	1.01 (0.20)	0.96 (0.27)	0.36
GT (IU/L)	241.40 (121.84)	265.44 (181.37)	0.57
BUN (mg/dL)	9.77 (5.69)	8.85 (4.47)	0.45
Serum creatinine (mg/dL)	0.61 (0.15)	0.63 (0.17)	0.72
Amylase (IU/L)	6.38 (2.98)	9.21 (4.44)	<0.05
CK (IU/L)	378.82 (123.24)	328.54 (172.66)	0.28
Ca (mg/dL)	9.03 (0.91)	8.74 (0.77)	0.18
Na (mEq/L)	138.20 (2.27)	137.62 (3.09)	0.42
K (mEq/L)	5.07 (0.66)	5.24 (0.98)	0.44
Cl (mEq/L)	105.30 (2.33)	105.13 (3.66)	0.84
Serum albumin (g/dL)	3.03 (0.37)	2.99 (0.38)	0.18

^a^Data are expressed as median (range).

The results of multiple logistic regression analysis are shown in Table 3. The number of days of theophylline administration from birth and hemoglobin levels were associated with an increased risk of adverse reactions after theophylline administration (p = 0.01, p = 0.03, respectively).

**Table 3:** Multivariate logistic regression analysis of factors associated with an increased risk of adverse reactions.

	Odds ratio (95% Cl)	p value
Days of theophylline administration from birth	1.77 (1.12–3.45)	0.01
Hemoglobin	0.25 (0.06–0.90)	0.03
Hematocrit	1.52 (0.98–2.53)	0.66
CK	1.00 (0.99–1.00)	0.29
Loading dose of theophylline	0.19 (0.03–1.02)	0.05
CL	0 (0–6.33e+24)	0.29

CK, creatine kinase; CL, total body clearance.

## Discussion

This study showed that theophylline is an effective prophylactic treatment of AOP. The incidence of effective treatment was 67.0%, and adverse reactions occurred in 21.0% of Japanese infants receiving mechanical ventilation. In addition, we demonstrated the clinical features of infants who received theophylline therapy for preventing AOP after weaning from mechanical ventilation, and evaluated predictive factors involved in the efficacy and safety of theophylline therapy.

Gestational age, body weight, and postconceptional age when theophylline administration was started were significantly lower in the ineffective group than in the effective group (Table 1). The underlying mechanism for AOP is thought to be immaturity of respiratory control with the incidence of AOP inversely proportional to gestational age [15, 16]. These results indicate that the efficacy of theophylline may be associated with prematurity in infants. Furthermore, in our study, multiple logistic regression analysis showed that gestational age was a predictive factor associated with an ineffective outcome, and the ROC curve demonstrated a gestational age cut-off value of 31.1 weeks (Fig 2). Therefore, physicians need to be aware of the possibility that theophylline fails to produce therapeutic effects for extubation in infants aged younger than 31.1 weeks old.

To confirm the possible involvement of theophylline concentrations in efficacy of treatment, we simulated concentration curves and calculated predicted concentrations, such as Cmax and the AUC. There was no significant difference in predicted concentrations of theophylline between the effective and ineffective groups (S1 Fig). As reported previously, a plasma theophylline concentration of at least 5 mg/L is necessary to produce therapeutic effects [17]. Despite theophylline being ineffective at a rate of 33%, predicted concentrations, even Cmin, were greater than 5 mg/L in this study. These findings suggest that theophylline concentrations may not be involved in preventing AOP when weaning from mechanical ventilation. Theophylline is extensively metabolized in premature infants and its major metabolic product is caffeine [18]. The demethylation pathway that predominantly occurs in adults is substituted by N-methylation to caffeine in premature infants. Caffeine and theophylline are comparably effective in AOP [19]. Therefore, caffeine might have affected the outcome of efficacy in our study.

In this study, multiple logistic regression analysis showed that the number of days of theophylline administration from birth was associated with an increased risk of adverse reactions (Table 3). This result suggests that the earlier the time of theophylline administration is, the more likely adverse reactions are.

The study was based on a retrospective review of medical records and has some limitations. First, the procedures for the management of neonates after extubation were not the same during the observation period. We did not examine some factors related to management, such as individual differences in attending physicians, nursing care, and medical devices. However, the procedures of prophylactic theophylline (aminophylline) for extubation in preterm infants did not change over the study period. Second, the definition of “effective” is not sufficiently objective. Because this study was retrospective, extubation or the use of theophylline was determined by physicians with clinical decision-making. Requirements for reintubation and administration of doxapram have been used as indicators of efficacy of theophylline in postmarketing surveillance in Japan. Third, we included infants in whom physicians noted adverse reactions after the start of theophylline treatment. Although infants in our neonatal intensive care unit are observed by physicians and trained medical staff, no definitive methods of determination of adverse reactions have been defined. Fourth, the PK parameters in this study were only predicted values because theophylline concentrations in infants in this study were not measured. Therefore, we could not determine if the actual PK parameters were different between the effective versus ineffective groups and between the adverse reaction (+) versus adverse reaction (−) groups. Although we validated our prediction of theophylline Cmin, further study is warranted to examine the PK parameters, and effectiveness and safety of prophylactic theophylline (aminophylline) for extubation in preterm infants.

Consequently, there may have been unmeasured confounders in the associations that we observed. Further study is required to confirm these findings. A prospective study needs to be performed considering these limitations. This prospective study should investigate whether there is an association with proposed predicted factors involved in the efficacy and safety of theophylline and prevention of AOP.

## Conclusion

In conclusion, we investigated the clinical features of infants who received theophylline therapy to prevent AOP after weaning from mechanical ventilation. We identified the predictive factors involved in the efficacy and safety of theophylline. Theophylline fails to produce therapeutic effects for extubation in infants aged younger than 31.1 weeks old, and adverse reactions can easily develop when theophylline is administered soon after birth.

## Supporting Information

S1 Fig Scatter plot of predicted concentrations of theophylline in the effective group and the ineffective group. (TIF)

S1 Table Validation of the minimum concentration of theophylline. (DOCX)
