# Supplementary figures and images for: Predictive Factors for Efficacy and Safety of Prophylactic Theophylline for Extubation in Infants with Apnea of Prematurity
Source: PLoS One. 2016 Jul 7;11(7):e0157198. doi: 10.1371/journal.pone.0157198 (PMC4936692; doi:10.1371/journal.pone.0157198)

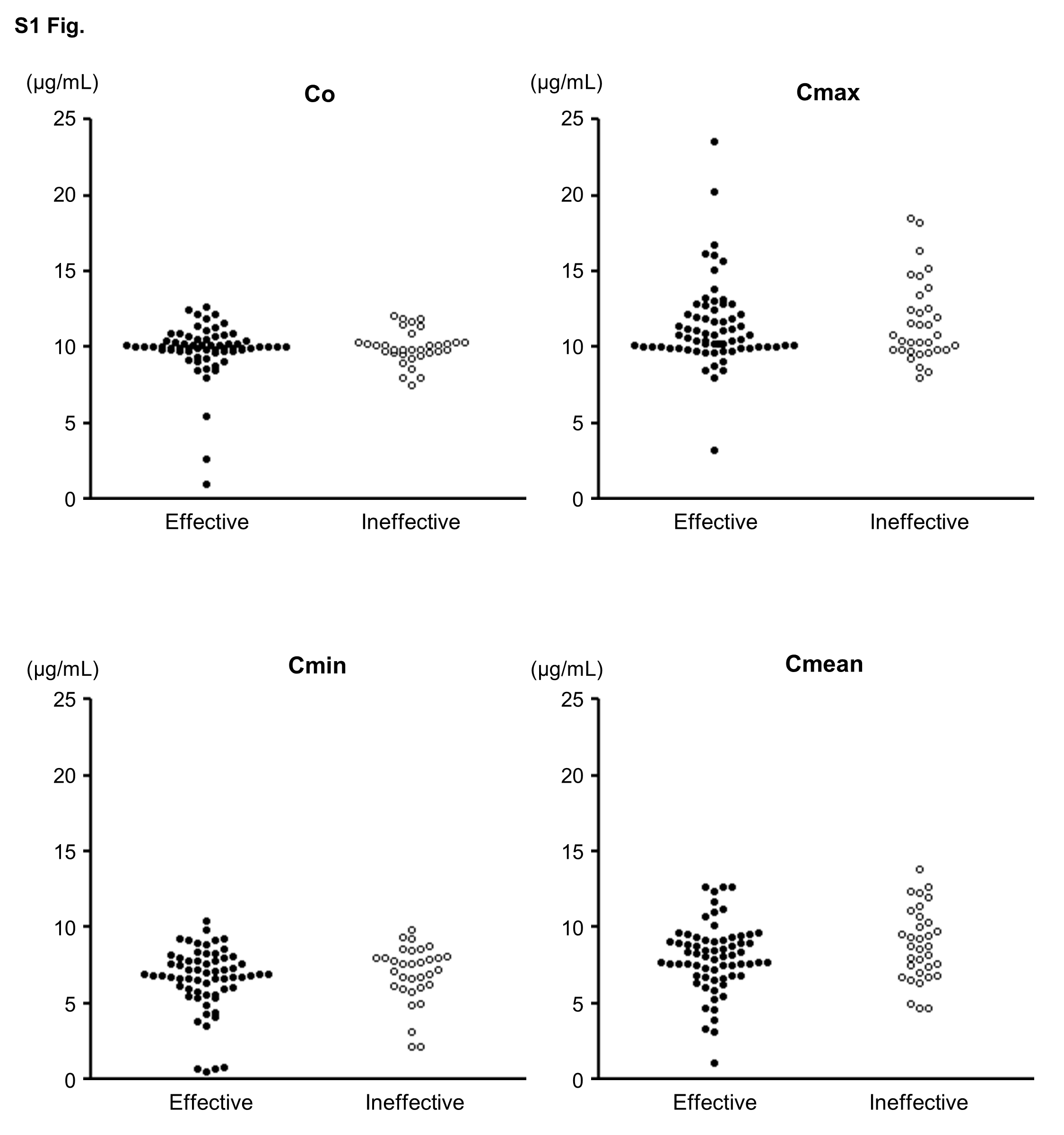

Supplement: S1 Fig — (TIF) [file pone.0157198.s001.tif]
