# Supplementary material for: Predictive Factors for Efficacy and Safety of Prophylactic Theophylline for Extubation in Infants with Apnea of Prematurity
Source: PLoS One. 2016 Jul 7;11(7):e0157198. doi: 10.1371/journal.pone.0157198 (PMC4936692; doi:10.1371/journal.pone.0157198)
Supplement: S1 Table — (DOCX) [file pone.0157198.s002.docx]

**S1 Table. Validation of the minimum concentration of theophylline**

|  | Mean prediction error  (μg/mL) | Mean absolute prediction error  (μg/mL) |
| --- | --- | --- |
| Mean | 1.10 | 1.27 |
| Standard deviation | 1.12 | 0.87 |
| 95% Confidence Interval | 0.12 - 2.09 | 0.51 - 2.04 |
